# Prevention of calpain-dependent degradation of STK38 by MEKK2-mediated phosphorylation

**DOI:** 10.1038/s41598-019-52435-8

**Published:** 2019-11-05

**Authors:** Atsushi Enomoto, Takemichi Fukasawa, Hiroki Tsumoto, Masataka Karube, Keiichi Nakagawa, Ayumi Yoshizaki, Shinichi Sato, Yuri Miura, Kiyoshi Miyagawa

**Affiliations:** 1 Laboratory of Molecular Radiology, Center for Disease Biology and Integrative Medicine, Graduate School of Medicine, University of Tokyo, 7-3-1 Hongo, Bunkyo-ku, Tokyo 113-8655 Japan; 2 Department of Dermatology, Graduate School of Medicine, University of Tokyo, 7-3-1 Hongo, Bunkyo-ku, Tokyo 113-8655 Japan; 3 Research Team for Mechanism of Aging, Tokyo Metropolitan Institute of Gerontology, 35-2 Sakae-cho, Itabashi-ku, Tokyo 173-0015 Japan; 4 Department of Radiation Oncology, National Cancer Center Hospital, 5-1-1 Tsukiji, Chuo-ku, Tokyo 104-0045 Japan; 5 Department of Radiation Oncology, University of Tokyo Hospital, 7-3-1 Hongo, Bunkyo-ku, Tokyo 113-8655 Japan

**Keywords:** Biochemistry, Cancer, Cell biology

## Abstract

Serine-threonine kinase 38 (STK38) is a member of the protein kinase A (PKA)/PKG/PKC-family implicated in the regulation of cell division and morphogenesis. However, the molecular mechanisms underlying STK38 stability remain largely unknown. Here, we show that treatment of cells with either heat or the calcium ionophore A23187 induced STK38 degradation. The calpain inhibitor calpeptin suppressed hyperthermia-induced degradation or the appearance of A23187-induced cleaved form of STK38. An *in vitro* cleavage assay was then used to demonstrate that calpain I directly cleaves STK38 at the proximal N-terminal region. Deletion of the N-terminal region of STK38 increased its stability against hyperthermia. We further demonstrated that the MAPKK kinase (MAP3K) MEKK2 prevented both heat- and calpain-induced cleavage of STK38. MEKK2 knockdown enhanced hyperthermia-induced degradation of STK38. We performed an *in vitro* MEKK2 assay and identified the key regulatory site in STK38 phosphorylated by MEKK2. Experiments with a phosphorylation-defective mutant demonstrated that phosphorylation of Ser 91 is important for STK38 stability, as the enzyme is susceptible to degradation by the calpain pathway unless this residue is phosphorylated. In summary, we demonstrated that STK38 is a calpain substrate and revealed a novel role of MEKK2 in the process of STK38 degradation by calpain.

## Introduction

Serine/threonine kinase 38 (STK38), also known as nuclear Dbf2-related 1 (NDR1), is a member of the highly conserved NDR family^1,2^. NDR family kinases in *Saccharomyces cerevisiae* have distinct roles. For example, Cbk1 is involved in the control of cell morphology^3^, whereas Dbf2 regulates mitotic exit and cytokinesis^4^. Another member of this family in *Schizosaccharomyces pombe*, Orb6, functions in cell polarity and morphogenesis^5^. Four related kinases of the NDR family exist in mammals: large tumour suppressor (LATS)1, LATS2, STK38/NDR1, and STK38L/NDR2^1,2^. LATS1 and LATS2 control mitotic exit and genomic stability^6,7^. STK38 is highly abundant in the organs of the immune system, whereas STK38L is expressed in the gastrointestinal tract and brain^2,8^. STK38 is implicated in regulation of centrosome duplication and mitotic chromosome alignment^9,10^. We previously demonstrated that the STK38/CDC25A signalling module regulates the DNA damage-induced G2/M checkpoint^11^. Several STK38 modulators have been identified to date, including mammalian sterile 20-like 3 (MST3)^12^, Mps one binder 1/2 (MOB1/2)^13,14^, and glycogen synthase kinase 3 (GSK-3)^15^. However, little is known about the molecular mechanisms that control STK38 protein stability.

The mitogen-activated protein kinase (MAPK) cascades, including the major components MAPK, MAPK kinase (MAP2K), and MAPKK kinase (MAP3K), are conserved in eukaryotic cells^16^. MAP3Ks phosphorylate and activate MAP2Ks, which in turn phosphorylate MAPKs. Increasing evidence from biochemical and genetic analyses suggests that MAP3Ks link various extracellular stimuli to cytoplasmic and nuclear effectors by activating downstream MAPK pathways^17^. MEKK1, the first MAP3K identified on the basis of its homology with the *Saccharomyces cerevisiae* MAP3K STE11, functions as a MAP3K for the ERK pathway^18^. MEKK2 is widely expressed and potently activates the NF-κB and MAPK pathways^19,20^.

To elucidate the molecular mechanisms of STK38 stability, in the present study, we investigated the effects of cellular stressors on its protein expression level in LU99, HeLa, and COS-7 cells.

## Results

### Heat treatment reduces STK38 protein levels

**Figure 1:**
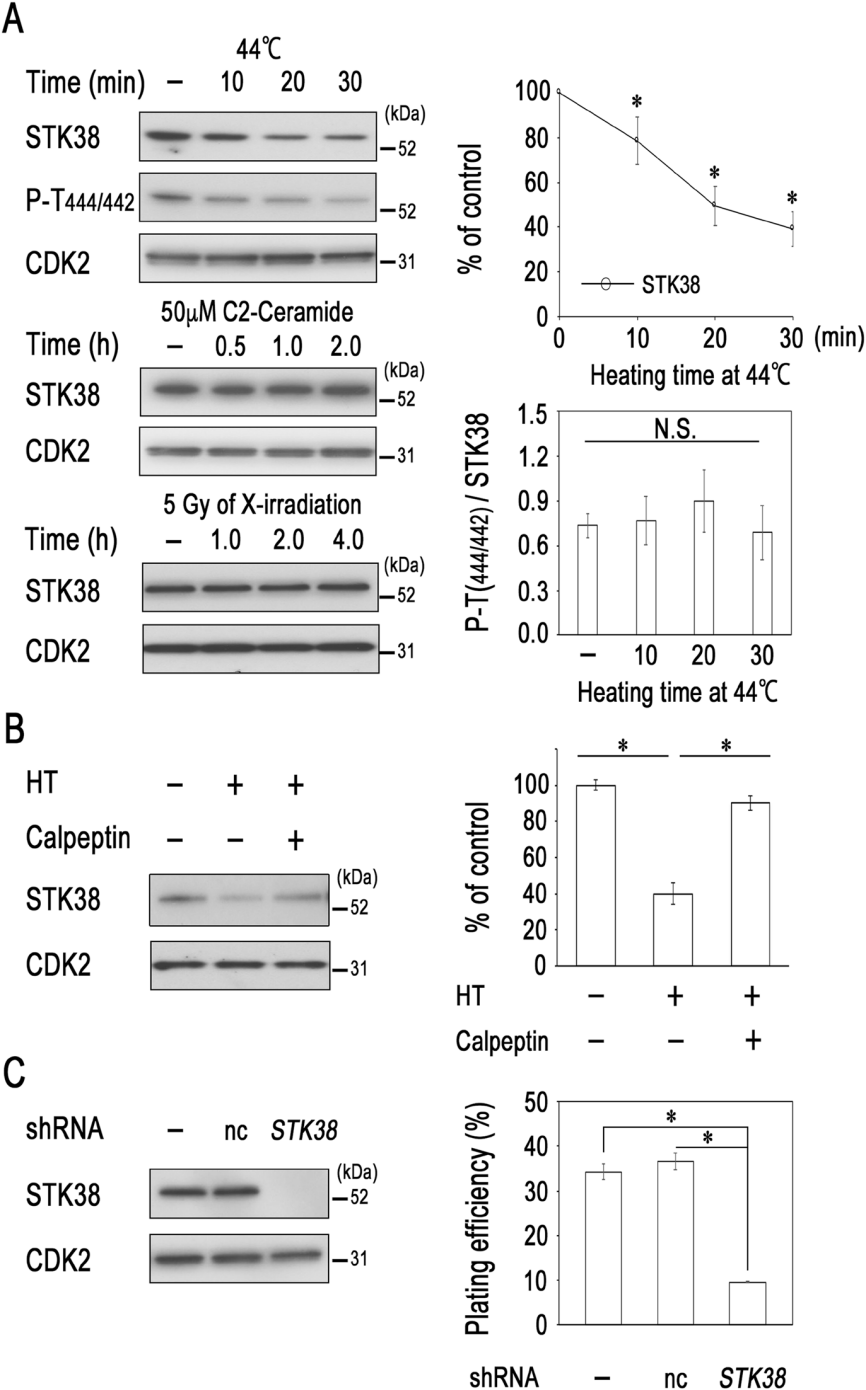
Hyperthermia decreases STK38 expression. (**A**) LU99 cells were heated to 44 °C (upper panel) or treated with 50 μM C2-ceramide (lower) for the indicated times. LU99 cells were irradiated with X-rays at 5 Gy and harvested at the indicated times (lower). (**B**) LU99 cells were pretreated with DMSO or 10 μM calpeptin for 1 h and then heated to 44 °C for 20 min. Cell lysates were prepared and analysed by western blotting with antibodies against the indicated proteins. CDK2 amount was used as loading control. A representative image with signal from immunoreactive STK38, phospho-Thr (444/442), or CDK2 is shown (see Supplementary Fig S4 for corresponding full-length image). Relative levels of STK38 or ratios of phospho-(Thr444/Thr442)/STK38 were determined from the western blot by using Image J software. Data are presented as the mean ± standard deviation of three independent experiments. Statistical significance was determined by the Student’s *t*-test (*P < 0.05). N. S., not significant. HT, heat treatment. (**C**) HeLa cells were transfected with a non-targeting control (nc) or *STK38*-specific shRNA expression vector; 24 h later, the cells were placed in culture medium containing 0.2 μg/mL puromycin and cultured for an additional 48 h. After the selection, the cells were assayed for plating efficiency by colony formation. Data are presented as the mean ± standard deviation of three independent experiments. Statistical significance was determined by the Student’s *t*-test (*P < 0.05).

We previously demonstrated that STK38 is activated by manipulations causing oxidative stress, such as X-ray irradiation or treatment with H_2_O_2_^11,15^. We further examined the effects of various stimuli on the expression and phosphorylation status of STK38 in human cancer cell lines and found that STK38 protein level decreased proportionally to the duration of hyperthermic treatment at 44 °C (Fig. 1A, upper panel). These results suggest that the decreased amount of STK38 after hyperthermia may be due to the instability of STK38 protein or the down-regulation of *STK38* gene expression. The level of STK38/STK38L hydrophobic motif phosphorylation at Thr-444/Thr-442, an indicator of kinase activity, was also decreased by hyperthermia. However, quantification of phospho-(Thr444/Thr442)/STK38 ratios by western blotting analysis indicated that this ratio did not significantly change by heat, suggesting that the level of both phospho- and total-STK38 is reduced by heat treatment. On the other hand, treatments with X-ray irradiation or C2-ceramide did not alter STK38 expression (Fig. 1A, lower panel).

Thermal stress generally causes protein unfolding. Cells deal with unfolded proteins by either refolding them with the help of molecular chaperones, or, if the protein structure cannot be rescued, by breaking them down^21^. Protein degradation can proceed through the ubiquitin-proteasome pathway and calpain degradation. We therefore examined which of these two pathways mediated heat-induced degradation of STK38. The addition of calpeptin, a calpain specific inhibitor, significantly suppressed heat-induced degradation of STK38 (Fig. 1B, left and right panels). Another calpain inhibitor, ALLN, partially reversed degradation of STK38 by heat treatment, but MG132, a commonly used 26S proteasome inhibitor, did not (Supplementary Fig. S1A). Our previous report demonstrated that Sp1 is necessary for the transcriptional regulation of the *STK38* promoter^22^. Thus, we assessed the effect of heat treatment on *STK38* transcriptional activity. As shown in Supplementary Fig. S1B, treatment with hyperthermia at 44 °C for 20–30 min did not affect *STK38* promoter activity. These findings suggested that reduction of STK38 observed in cells heating at 44 °C for the indicated times occurred due to its degradation by calpain pathway but not from the down-regulation of its transcription.

To clarify the biological significance of STK38 degradation, we conducted colony-formation assays to determine the effect of reduced STK38 expression on proliferation ability. Transfection with *STK38* short hairpin RNA (shRNA), but not with a control expression vector, specifically knocked down the endogenous STK38 expression in HeLa cells (Fig. 1C, left panel). The plating efficiency decreased markedly in the *STK38* shRNA-expressing HeLa cells compared to parental HeLa cells or those expressing control shRNA (Fig. 1C, right panel). These results suggest that STK38 might play an important role in cell proliferation.

### Cleavage of STK38 by calpain

**Figure 2:**
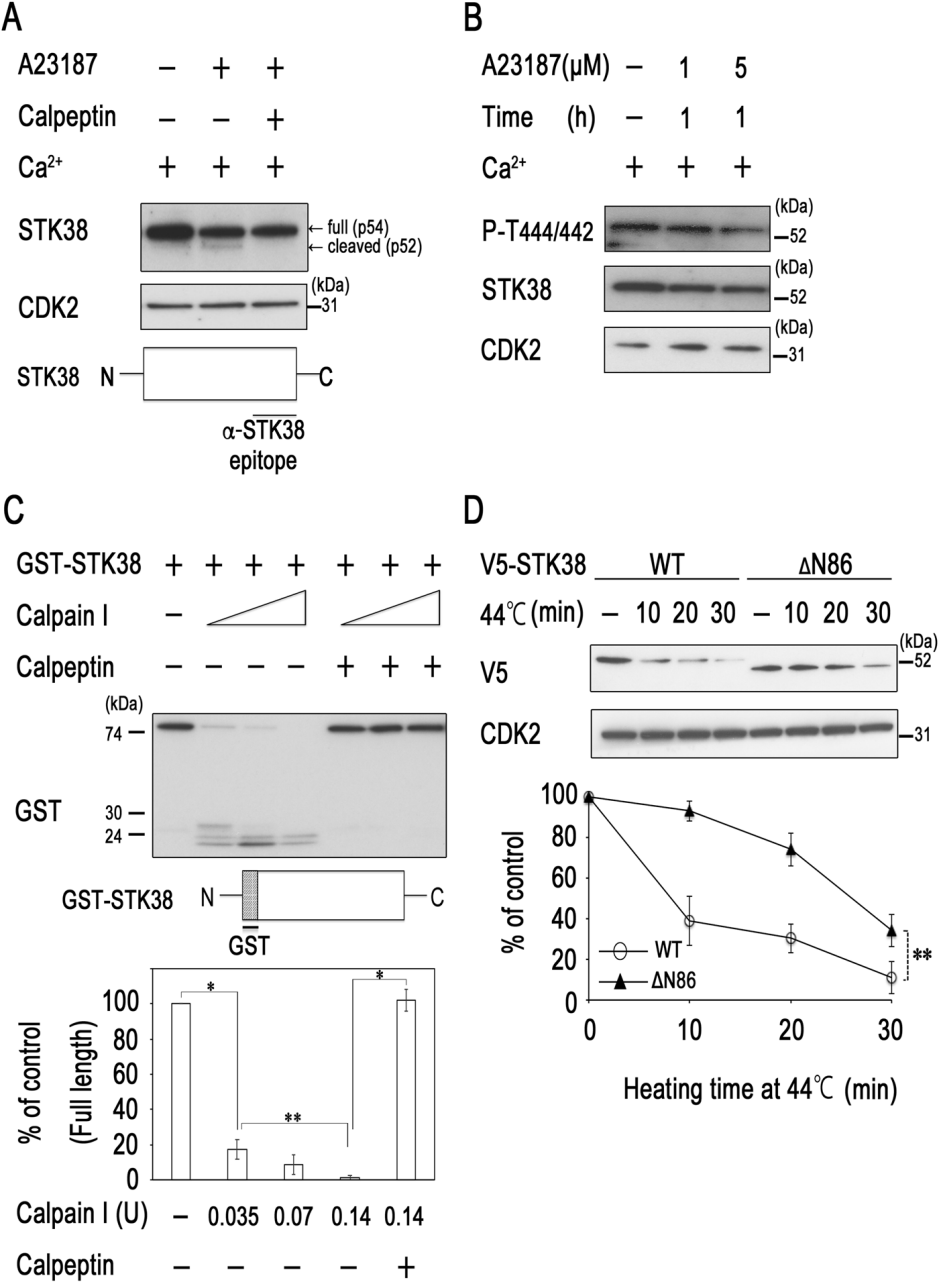
Cleavage of STK38 by calpain. (**A**,**B**) HeLa cells were treated with 1–5 μM A23187 in the absence or presence of 10 μM calpeptin for 1 h. (**C**) *In vitro* STK38 cleavage assay. GST-tagged STK38 (GST-STK38) was incubated with different concentrations of calpain I (0.035, 0.07, 0.14 units of calpain I in lanes 2–4 and 6–8, respectively) in the absence or presence of 10 μM calpeptin for 15 min at 30 °C. (**D**) The N-terminus of STK38 contains thermosensitive regions. COS-7 cells were transiently transfected with a mammalian expression vector encoding either V5-tagged murine full-length or ΔN STK38 (87–465). Forty-eight hours after transfection, the cells were heated to 44 °C for the indicated times or left untreated. The *in vitro* reaction products or cell lysates were analysed by western blotting with antibodies against the indicated proteins. A representative image of western blot is shown (see Supplementary Fig S5 for corresponding full-length image). Relative levels of STK38 were determined from western blots by using Image J software. Data are presented as the mean ± standard deviation of three independent experiments. Statistical significance was determined by the Student’s *t*-test (*P < 0.005; **P < 0.05). Schematic of epitope location of STK38 antibody (2F6) or the N-terminal GST-STK38 protein is presented.

Hyperthermia triggers endoplasmic reticulum (ER) stress or alters the permeability of plasma membranes, resulting in calcium spikes^21^. Thus, we next tested whether an increase in intracellular calcium decreased STK38 protein level. Immunoreactive proteins recognised by an anti-STK38 monoclonal antibody were mainly revealed as 54 kDa (p54) bands in western blots of HeLa cell extracts, as had been previously demonstrated in many other mammalian cell lines^15^. However, we found an additional band of 52 kDa (p52) after treatment of HeLa cells with the calcium ionophore A23187 (Fig. 2A). Addition of calpeptin blocked the conversion of p54 to p52, suggesting that p52 is a cleaved form of STK38. Moreover, the analysis of molecular weight of the cleaved fragments detected by the anti-STK38 monoclonal antibody that recognises a C-terminus epitope suggested that cleavage site of A23187-stimulated protease is at the N-terminus of STK38. On the other hand, cleaved isoform p52 of STK38 was not detected after heat treatment (Fig. 1A). Recently, SOCS2 (suppressor of cytokine signalling 2), one of the substrate recognition modules of Cullin5/Rbx2 ubiquitin ligases, was demonstrated to interact with STK38 and promote its ubiquitin-mediated degradation^23^. Thus, STK38 seems to be subjected to proteolysis through several pathways. Heat may induce degradation of STK38 in different manner of A23187 or through multiple pathways which would decrease p52 amount to undetectable levels. We also found that A23187 decreased the level of STK38/STK38L phosphorylation at Thr-444/Thr-442 in a dose-dependent manner (Fig. 2B). However, quantification of phospho-(Thr444/Thr442)/STK38 ratios by western blotting analysis indicated that this ratio did not significantly change by A23187 (Supplementary Fig. S1C).

Calpains are calcium-activated neutral proteases that catalyse the cleavage of a wide variety of proteins, including enzymes, transcriptional factors, and cytoskeletal proteins, in many mammalian tissues^24,25^. To determine whether calpain directly cleaves STK38, we incubated purified calpain I with N-terminally GST-tagged STK38. The amount of the full-length STK38 dose-dependently and significantly decreased after addition of calpain I, which coincided with the appearance of its cleaved fragments (Fig. 2C). Calpeptin completely blocked calpain I-mediated cleavage of STK38. The analysis of molecular weight of the cleaved fragments detected by an anti-GST antibody indicated that STK38 was cleaved at the calpain I-sensitive sites in the N-terminal region. Using the computational tool GSP-CCD1.0 (http://ccd.biocuckoo.org/down.php), most of putative calpain cleavage sites were mapped to the N-terminal region of STK38. To further determine whether the N-terminus of STK38 confers heat sensitivity, we investigated the effect of the N-terminal deletion (Δ1–86) on thermal stability. We found that the removal of the N-terminal region of murine STK38 significantly decreased its degradation after hyperthermia (Fig. 2D). We also attempted to identify PEST sequences, built-in signals responsible for rapid protein degradation, in STK38 by using the PESTFind algorithm (https://emboss.bioinformatics.nl/cgi-bin/emboss/epestfind) and revealed six PEST motifs in STK38. However, these sequences had characteristics of poor PEST motifs (PEST scores <5) and were located in the middle of the protein or in the C-terminal regions. Those results together with our present data indicated that the proximal N-terminal region of STK38 is the target of calpain, but PEST motifs are probably not required for calpain- and heat-mediated degradation of STK38.

### MEKK2 inhibits heat-induced degradation of STK38

**Figure 3:**
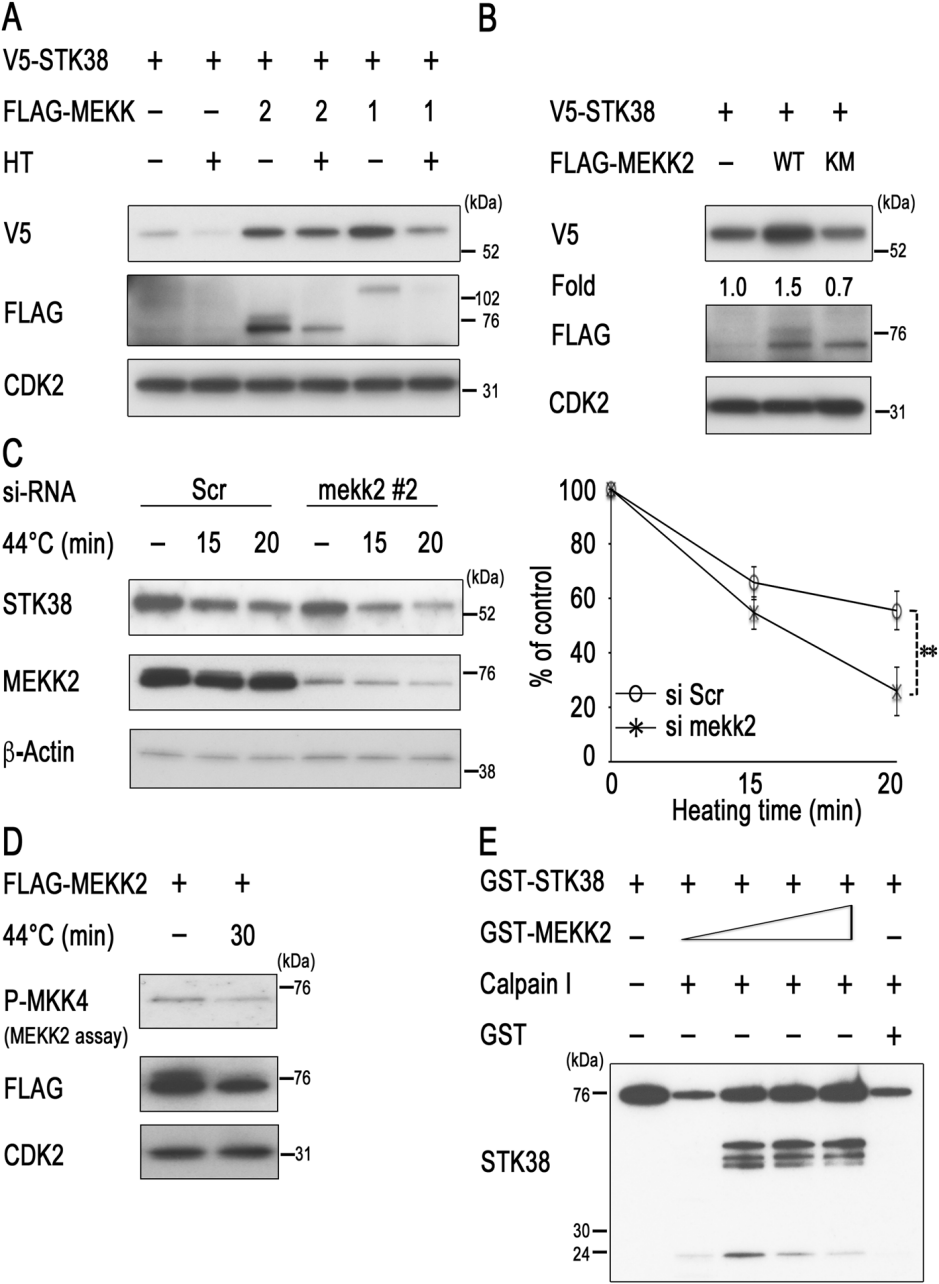
MEKK2 inhibits heat- and calpain-mediated degradation of STK38. (**A**) COS-7 cells were transfected with human STK38-V5 alone, or with FLAG-MEKK1 or FLAG-MEKK2. Forty-eight hours after transfection, the cells were heated to 44 °C for 20 min or left untreated as controls and harvested. (**B**) COS-7 cells were transfected with human STK38-V5 alone, or with FLAG-MEKK2 (WT) or FLAG-MEKK2 (KM). Forty-eight hours after transfection, the cells were harvested. (**C**) HeLa cells were transfected with the scramble oligonucleotides control (scr) or *MEKK2*-specific siRNA. Forty-eight hours after transfection, the cells were heated to 44 °C for the indicated times or left untreated as controls and harvested. (**D**) HeLa cells were transfected with FLAG-MEKK2. Forty-eight hours after transfection, the cells were heated to 44 °C for the indicated times or left untreated as controls and harvested. The MEKK2 activity was measured by immune complex kinase assay with an anti-FLAG antibody using GST-MKK4 as the substrate. (**E**) GST-STK38 was incubated with calpain I (0.07 units of calpain I in each lane) in the absence or presence of GST-active MEKK2 for 15 min at 30 °C. Cell lysates or *in vitro* reaction products were analysed by western blotting with the antibodies against the indicated proteins. A representative image of western blot is shown (see Supplementary Fig S6 for corresponding full-length image). Relative levels of STK38 were determined from the western blot using Image J software. Data are presented as the mean ± standard deviation of three independent experiments. Statistical significance was determined by the Student’s *t*-test (**P < 0.05).

Our previous report demonstrated that STK38 interacts with MAP3Ks and is involved in regulating MAPK signalling pathways^26^. Therefore, we examined a possible involvement of MAP3Ks in the regulation of STK38 stability. We first investigated whether heat-induced STK38 protein instability was influenced by the co-transfection of MAP3Ks in COS-7 cells. Interestingly, overexpression of MEKK1/2 enhanced STK38 expression. MEKK2 also rescued heat-induced instability of STK38, but MEKK1 did not (Fig. 3A). To ascertain that *STK38* gene transcription was not enhanced, the relative STK38 promoter activity was analysed with and without MEKK2. Overexpression of MEKK2 did not stimulate of STK38 promoter activity (Supplementary Fig. S2A), suggesting that the increased expression of STK38 by MEKK2 may be mediated by post-translational modification but not through transcriptional changes. We also analysed the effects of a kinase-inactive mutant MEKK2 (KM) on the expression level of STK38 protein. We found that wild-type MEKK2 increased STK38 protein level, whereas kinase-inactive mutant MEKK2 did not (Fig. 3B). These results suggest that MEKK2 activity may be necessary for the regulation of STK38 protein stability. To confirm that MEKK2 acts as a positive regulator of STK38, we examined the effects of *MEKK2* knockdown on STK38 protein stability. Transfection with two different siRNAs against *MEKK2* (#2 and #3) knocked down expression of the endogenous MEKK2 protein and significantly enhanced heat-induced degradation of STK38 protein without affecting *STK38* mRNA levels, respectively (Fig. 3C; Supplementary Fig. S2B,C). These results indicate that enhanced suppression of STK38 was not due to an off-target effect of siRNA and suggest that MEKK2 protects STK38 from heat-induced degradation. Interestingly, treatment with heat also reduced MEKK2 activity (Fig. 3D). Moreover, we examined whether MEKK2 directly affects calpain-mediated cleavage of STK38 by the *in vitro* cleavage assay. MEKK2 significantly rescued calpain I-mediated cleavage of STK38, and this treatment led to the appearance of its several intermediate fragments (Fig. 3E, Supplementary Fig. S2D). These results suggest that MEKK2 prevents STK38 from calpain-dependent degradation.

### Phosphorylation of Ser91 by MEKK2 contributes to STK38 stability

**Figure 4:**
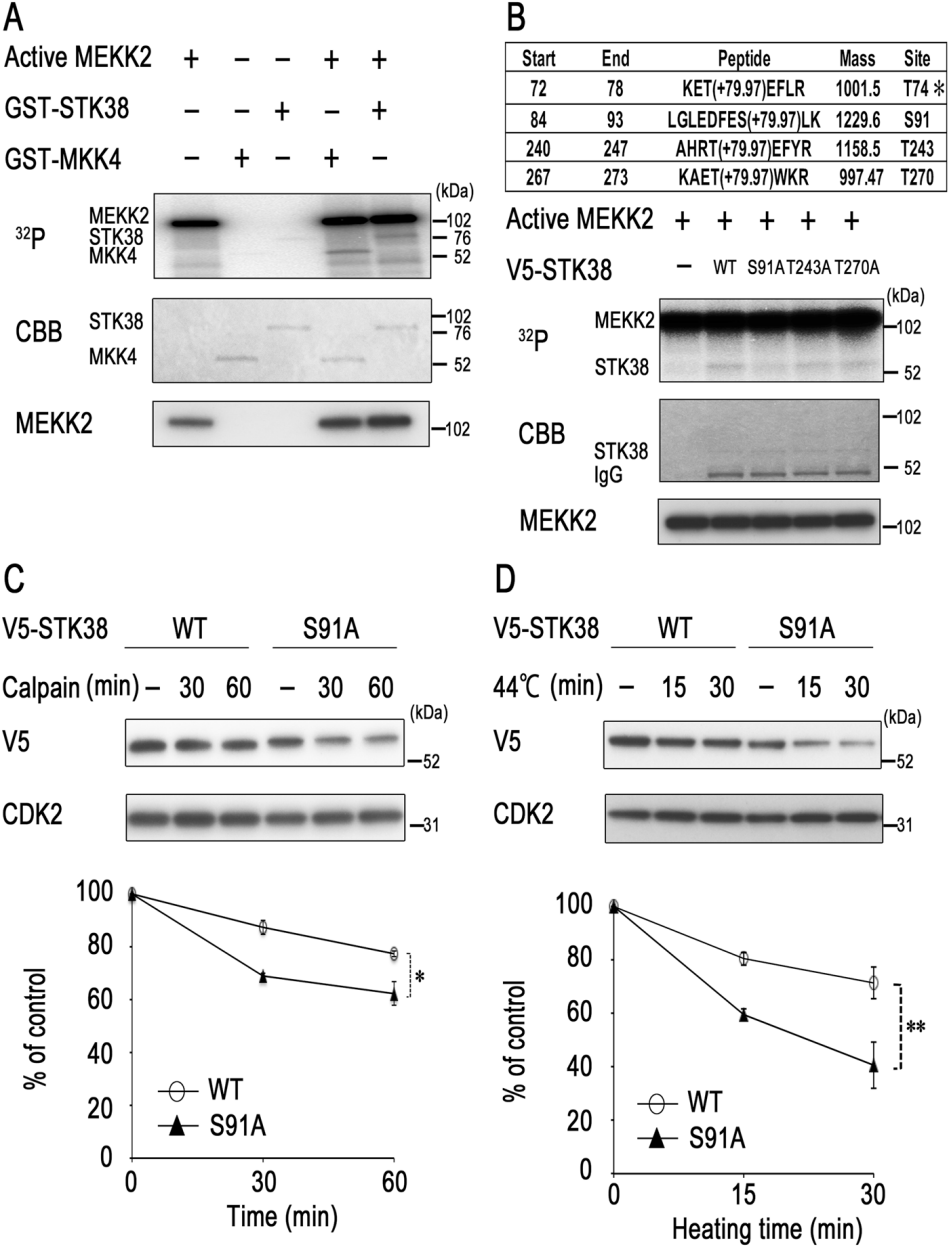
Maintenance of STK38 stability requires its phosphorylation. (**A**) MEKK2 phosphorylates STK38 *in vitro*. GST-tagged STK38 (unactive) or MKK4 (unactive) was incubated with or without active MEKK2 in the presence of [γ-^32^P] ATP for 30 min at 30 °C. (**B**, upper) Putative phosphorylation sites identified within STK38. Inactive GST-STK38 was incubated with or without active MEKK2, and the kinase reaction products were subjected to SDS-PAGE. GST-STK38 was excised and processed by tryptic cleavage for MS analysis. *T74 is endogenously phosphorylated. (**B**, bottom) *In vitro* kinase reaction was performed by incubating active MEKK2 alone or with the V5-immunopurified wild-type STK38, STK38 (S91A), STK38 (T243A), STK38 (T270A) from the transfected 293 T cells for 15 min at 30 °C. The kinase reaction products were subjected to SDS-PAGE and then visualised by autoradiography (^32^P, top panel) or Coomassie Brilliant Blue staining (CBB, bottom panel). (**C**) COS-7 cells were transfected with human STK38-V5 (WT) or STK38-V5 (S91A). Forty-eight hours after transfection, the cells were harvested. *In vitro* cleavage reaction was performed by incubating calpain I (0.07 units) with lysates (30 μg) of the transfected cells from STK38-V5 (WT) or STK38-V5 (S91A) for 30–60 min at 30 °C. Reaction products were subjected to western blotting analysis with antibodies against the indicated proteins. (**D**) COS-7 cells were transfected with human STK38-V5 (WT) or STK38-V5 (S91A). Forty-eight hours after transfection, the cells were treated as described in Fig. 2D. Cell lysates were analysed by western blotting with antibodies against the indicated proteins. A representative image of western blot, CBB-stained gel, or autoradiography is shown (see Supplementary Fig S7 for corresponding full-length image). Relative levels of STK38 were determined from the western blot by using Image J software. Data are presented as the mean ± standard deviation of three independent experiments. Statistical significance was determined by the Student’s *t*-test (*P < 0.05; **P < 0.01).

To determine whether the action of MEKK2 on STK38 stability is mediated by MEKK2-catalysed STK38 phosphorylation, we performed the *in vitro* kinase assay. GST-tagged inactive STK38 was mixed with GST-tagged active MEKK2 in the presence of [γ-^32^P] ATP. As shown in Fig. 4A, MEKK2 phosphorylated STK38 as well as MKK4, a well-known substrate of MAP3K. These findings prompted us to map the phosphorylation sites in STK38 that are necessary for regulation of its stability. Approximately 1 μg of GST-STK38 treated with active MEKK2 was digested by trypsin, and the resultant mixture of peptides was analysed by nanoLC-MS/MS. Identified proteins, peptides, phosphorylated peptides of STK38 are summarised in Supplementary Tables S1, S2, and Fig. S3, respectively. As a result, two known and two novel sites for MEKK2 phosphorylation were found in human STK38 sequence, namely, Thr74, Ser91, Thr243, and Thr270 (Fig. 4B, upper part). Phosphorylation of Ser91, Thr243, and Thr270 was detected in the presence of MEKK2, but that of Thr74 was also identified in the absence of MEKK2, indicating that the latter was endogenously phosphorylated (Supplementary Fig. S3). Each of the former three residues was converted to alanine by site-directed mutagenesis, and V5-tagged human STK38 wild-type and mutant proteins were expressed in HEK 293 T cells and immunopurified by an anti-V5 antibody. To determine whether these amino acids are phosphorylated by MEKK2, we performed an *in vitro* MEKK2 assay using immunopurified STK38 variants as substrate and found that phosphorylation of STK38 with the S91A mutation was markedly lower than that of wild-type, T243A, or T270A mutant protein (Fig. 4B, lower part). These results indicated that STK38 undergoes phosphorylation on Ser91 by MEKK2 *in vitro*.

Phosphorylation, as a common protein modification, regulates protein stability, but its role in protein degradation is different^27,28^. Phosphorylation of some proteins triggers proteasome- or calpain-dependent degradation, whereas phosphorylation of other proteins protects them from proteolysis^28–30^. To investigate the significance of STK38 phosphorylation by MEKK2, we examined susceptibility of STK38 variants to calpain-mediated degradation by the *in vitro* cleavage assay. After addition of calpain I, the S91A mutant was more rapidly degraded than the wild-type protein, indicating that the former was more sensitive to calpain (Fig. 4C). We further investigated thermal stability of STK38 variants. COS-7 cells were transiently transfected with constructs expressing either V5-tagged human wild-type STK38 or S91A mutant and then subjected to heat treatment. After hyperthermia, the S91A mutant was degraded more rapidly than the wild-type protein (Fig. 4D). Moreover, S91A mutant expression level was lower than that of the wild-type STK38, indicating that Ser91 is an important residue for STK38 stability. Our data suggest that Ser91 phosphorylation of STK38 by MEKK2 possibly blocks the interaction of calpain with STK38 or disrupts proper conformation for cleaving, thereby protecting STK38 from calpain-dependent degradation.

## Discussion

We have shown that STK38 has a heat-sensitive region in the N-terminus and is degraded by the calpain pathway (Fig. 2). STK38 and related proteins have a conserved N-terminal regulatory domain, a catalytic domain, and a C-terminal regulatory domain. The N-terminal regulatory domain of the STK38 family includes a number of conserved basic hydrophobic residues and is predicted to form an amphiphilic α-helix. Several modulator proteins, including Mob1/2, associate with the N-terminal regulatory domain of STK38 and stimulate its kinase activity^13,14^. Thus, calpain-mediated cleavage of STK38 likely disrupts its association with the modulators and thereby inhibits its full activation. However, STK38 can be stabilised as a result of phosphorylation at Ser91 by MEKK2. This destabilisation seems to occur in particular when MEKK2 is inactivated. Phosphorylated MEKK2 bands migrate slower than those of non- or hypophosphorylated bands, due to MEKK2 autophosphorylation^20,26^. Interestingly, hyperthermia decreased the levels of both slower and faster migrating bands (Fig. 3A, lanes 3 and 4) and attenuated MEKK2 activity (Fig. 3D), suggesting that MEKK2 is inactivated by heat. On the other hand, our results indicated that inactivation of MEKK2 by the transfection of its dominant negative form did not stimulate STK38 stability (Fig. 3B). Moreover, knockdown of MEKK2 by introducing corresponding siRNA reduced STK38 stability (Fig. 3C). Together, these findings suggest that heat-induced inactivation of MEKK2 decreases the level of STK38 phosphorylation at Ser91 and thereby impairs its stability. Future work aimed at clarifying the regulatory mechanism of MEKK2 stability will be necessary to completely understand the process of STK38 degradation.

The available experimental evidence suggests that STK38 acts as an oncogene or a co-dependent regulator thereof. It was shown that overexpression of STK38 potentiated sphere-forming capacity in PC3 cells^23^. STK38 regulates Myc protein stability, and STK38 knockdown suppresses growth of Myc-addicted tumors *in vivo*^31^. We also observed that STK38 knockdown decreased plating efficiency of HeLa cells (Fig. 1C). Together, our results indicate that thermal stress decreases STK38 stability via negative impact on MEKK2 and raise the potential of STK38 as a target in hyperthermia.

## Materials and Methods

### Cell culture, transfection, and stimulation

HeLa and LU99 cells were purchased from Japanese Collection of Research Bioresources Cell Bank (Ibaraki, Osaka). HEK293T and COS-7 cells were gifts from Prof. Katsuji Yoshioka (Kanazawa University). HEK293T, HeLa, COS-7, and LU99 cells were cultured as described previously^15,26^. Transient transfections were performed using FuGENE HD (Roche, Indianapolis, IN) and Lipofectamine 2000 (Takara Bio, Shiga, Japan) as described previously^26^. Hyperthermic treatment was carried out by submerging the culture flask in a water bath (Ikemoto Rika, Tokyo, Japan) set at 44 °C with a precision of ± 0.05 °C. LU99 cells were pretreated with MG132 (Sigma, St. Louis, MO), ALLN (Sigma), or calpeptin (Calbiochem, Darmstadt) at 5–10 μM for 60 min, and then stimulated for the indicated times with hyperthermic treatment. To investigate the effect of calcium ionophore on STK38 stability, HeLa cells were treated with 1–5 μM A23187 in PBS buffer containing 1 mM CaCl_2_ (Cayman Chemical, Ann Arbor, MI) or vehicle for 1 h, and then harvested. Treatment of cells with X-irradiation or C2-ceramide was carried out as described previously^15,22^. To determine the plating efficiency (PE) of STK38-knockdown cells, HeLa cells were transiently transfected with either a non-targeting or an *STK38*-specific shRNA expression vector, then selected for 48 h in medium containing 0.2 μg/mL puromycin (Invivogen, San Diego, CA). After selection, the cells were trypsinised, diluted, counted, and seeded into 60-mm dishes at various cell densities. After 14 days, the colonies were stained with crystal violet, and those containing more than 50 cells were counted.

### Western blot analysis

Western blot analysis was performed as described previously^26^. The blots were then incubated with one of the following antibodies: anti-STK38 (2F6, Abnova, Jhouzih St., Taipei), anti-phospho-NDR1/2 (Thr 444/442, Signalway Antibody, College Park, MD), anti-CDK2 (Santa Cruz Biotechnology, Santa Cruz, CA), anti-MEKK2 (Epitomics, Burlingame, CA), anti-phospho-MKK4 (Cell Signaling Technology, Beverly, MA), anti-V5 (Nacalai Tesque, Kyoto, Japan), anti-FLAG (M2, Sigma), anti-GST (Santa Cruz Biotechnology), or anti-β-actin (Sigma). The captured images were analysed with Image J image-processing software and quantified by measuring the density of each protein band.

### *In vitro* proteolytic cleavage assay

The cleavage reactions were initiated by the addition of 0.035–0.14 units of calpain I (Calbiochem) to 200 ng of the purified GST-tagged STK38 with or without 100 ng of active MEKK2 in a total volume of 20 μL of the cleavage buffer (20 mM HEPES, pH 7.5, 50 mM KCl, 2 mM MgCl_2_, 5 mM CaCl_2_, 1 mM DTT). The cleavage reactions were incubated for 0.5 h at 30 °C and were stopped by the addition of 2 × SDS-sample buffer. They were then analysed by western blotting using an anti-GST antibody.

### Immunoprecipitation and *in vitro* kinase assays

Immunoprecipitation was performed as described previously^26^. For the MEKK2 kinase assay, V5-STK38 immunoprecipitates were incubated with 10 ng of active-MEKK2 (Signal Chem, Richmond, BC) in MEKK2 kinase buffer containing 0.37 MBq ml^−1^ [γ-^32^P] ATP at 30 °C for 15 min. For in *vitro* kinase assays, active MEKK2 was incubated with 1.0 μg of wild-type inactive STK38 (Signal Chem) or inactive MKK4 (Signal Chem) in MEKK2 kinase buffer containing 0.37 MBq ml^−1^ [γ-^32^P] ATP for 30 min at 30 °C. The kinase reaction products were subjected to SDS-PAGE and analysed with a phosphoimaging device (BAS-2000; GE Healthcare, Buckinghamshire, UK).

### In-gel digestion of proteins with trypsin

GST-STK38 preparations treated with or without active MEKK2 were separated by SDS-PAGE in 10% gel, stained with Coomassie Blue R-350, and excised from the gel. The gel pieces were destained twice with 50% acetonitrile in 25 mM NH_4_HCO_3_ at 37 °C for 30 min with shaking, reduced with 50 mM dithiothreitol in 25 mM NH_4_HCO_3_ at 60 °C for 10 min, alkylated with 100 mM iodoacetamide in 25 mM NH_4_HCO_3_ at room temperature (RT) for 1 h in the dark, washed twice with 50% acetonitrile in 25 mM NH_4_HCO_3_ at 37 °C for 15 min with shaking, dehydrated with acetonitrile at RT for 15 min, and allowed to air-dry for 10 min. The dried gel pieces were treated with trypsin in 25 mM NH_4_HCO_3_ at 37 °C for 17 h. The cleaved peptides were extracted with 1% trifluoroacetic acid and desalted using a MonoSpin C18 spin column (GL Sciences, Tokyo, Japan). The eluted peptides were reconstituted in 0.1% formic acid (FA) and subjected to nanoLC-MS/MS.

### Nano liquid chromatography tandem mass spectrometry (nanoLC-MS/MS)

For the identification of phosphorylation sites, nanoLC-MS/MS was performed on an UltiMate 3000 RSLCnano system (Thermo Fisher Scientific) coupled to a Q Exactive hybrid quadrupole-Orbitrap mass spectrometer (Thermo Fisher Scientific) equipped with nano ESI source. The nanoLC system was equipped with a trap column (C18 PepMap 100, 0.3 mm × 5 mm, 5 μm, Thermo Fisher Scientific) and an analytical column (NTCC-360/75-3-125, Nikkyo Technos, Tokyo, Japan). Peptides separation was performed using 60 min gradient of water containing 0.1% FA (mobile phase A) and acetonitrile containing 0.1% FA (mobile phase B) at a flow rate of 300 nL/min. The elution gradient was set as follows: 0–3 min, 2–2% B; 3–63 min, 2–40% B; 63–65 min, 40–95% B; 65–75 min, 95–95% B; 75–77 min, 95–2% B; 77–90 min, 2–2% B. Mass spectrometer was operated in data-dependent acquisition mode.

### Protein identification

All MS/MS data were analysed by PEAKS Studio X software (Bioinformatics Solutions Inc., Waterloo, ON, Canada).

### Plasmids and siRNAs

The expression vectors for FLAG-tagged MEKK1, FLAG-MEKK2, V5-tagged STK38, and V5-ΔN STK38 were previously described^26^. Site-directed mutagenesis was performed using a Prime STAR Mutagenesis kit (Takara Bio). The sequences of the ORFs in the constructed plasmids were confirmed by DNA sequencing. The mammalian *STK38* shRNA expression vector was described previously^26^. Synthetic siRNA duplex oligonucleotides specific for regions in human *MEKK2 (MAP3K2)* mRNA were designed and synthesised by Invitrogen (Carlsbad, CA). The target sequences were as follows (only the antisense sequence is shown).

Stealth siRNA *MEKK2* #662: 5′-GGAACUGCUGGAUCGUAGUAUUCAU-3′.

Stealth siRNA *MEKK2* #663: 5′-CCAAUAACGAGUUGGUAAUUCCAUU-3′.

### Semi-quantitative RT-PCR analysis

RNA extraction, cDNA synthesis and Semi-quantitative RT-PCR were performed as described previously^22^.

### Reporter assay

COS-7 or HEK293T cells were plated onto 24-well plates at a density of 1 × 10^4^ cells/well, 1 day prior to transfection. The cells were transfected with 50 ng pRL (*Renilla* luciferase)-SV40 and 1.0 μg pGL3 (*Firefly* luciferase) reporter plasmids containing the *STK38* promoter with or without pcD3.1 FLAG-MEKK2. Twenty-four hours after transfection, the cells were either treated with heat or left untreated. Cell extracts were prepared and luciferase activity was measured, as described previously^22^.

## Supplementary information

Supplementary information

Supplementary information

## Data Availability

The datasets produced and/or analysed during this study are available from the corresponding author on reasonable request.
