# Supplementary material for: Prevention of calpain-dependent degradation of STK38 by MEKK2-mediated phosphorylation
Source: Sci Rep. 2019 Nov 5;9:16010. doi: 10.1038/s41598-019-52435-8 (PMC6831656; doi:10.1038/s41598-019-52435-8)
Supplement: Supplementary file 2 — Supplementary information [file 41598_2019_52435_MOESM2_ESM.pdf]

Supplementary Information

Prevention of calpain-dependent degradation of STK38 by MEKK2-mediated phosphorylation

Atsushi Enomoto[1*], Takemichi Fukasawa[1,2], Hiroki Tsumoto[3], Masataka Karube[4], Keiichi Nakagawa[5], Ayumi Yoshizaki[2], Shinichi Sato[2], Yuri Miura[3], and Kiyoshi Miyagawa[1]

[1]Laboratory of Molecular Radiology, Center for Disease Biology and Integrative Medicine, Graduate School of Medicine, University of Tokyo.

[2]Department of Dermatology, Graduate School of Medicine, University of Tokyo.

[3]Research Team for Mechanism of Aging, Tokyo Metropolitan Institute of Gerontology.

[4]Department of Radiation Oncology, National Cancer Center Hospital.

[5]Department of Radiation Oncology, University of Tokyo Hospital.

[*]To whom all correspondence should be addressed

E-mail: aenomoto-tky@umin.ac.jp

Contents: Supplementary Figures S1-S7

Supplementary Tables S1 and S2

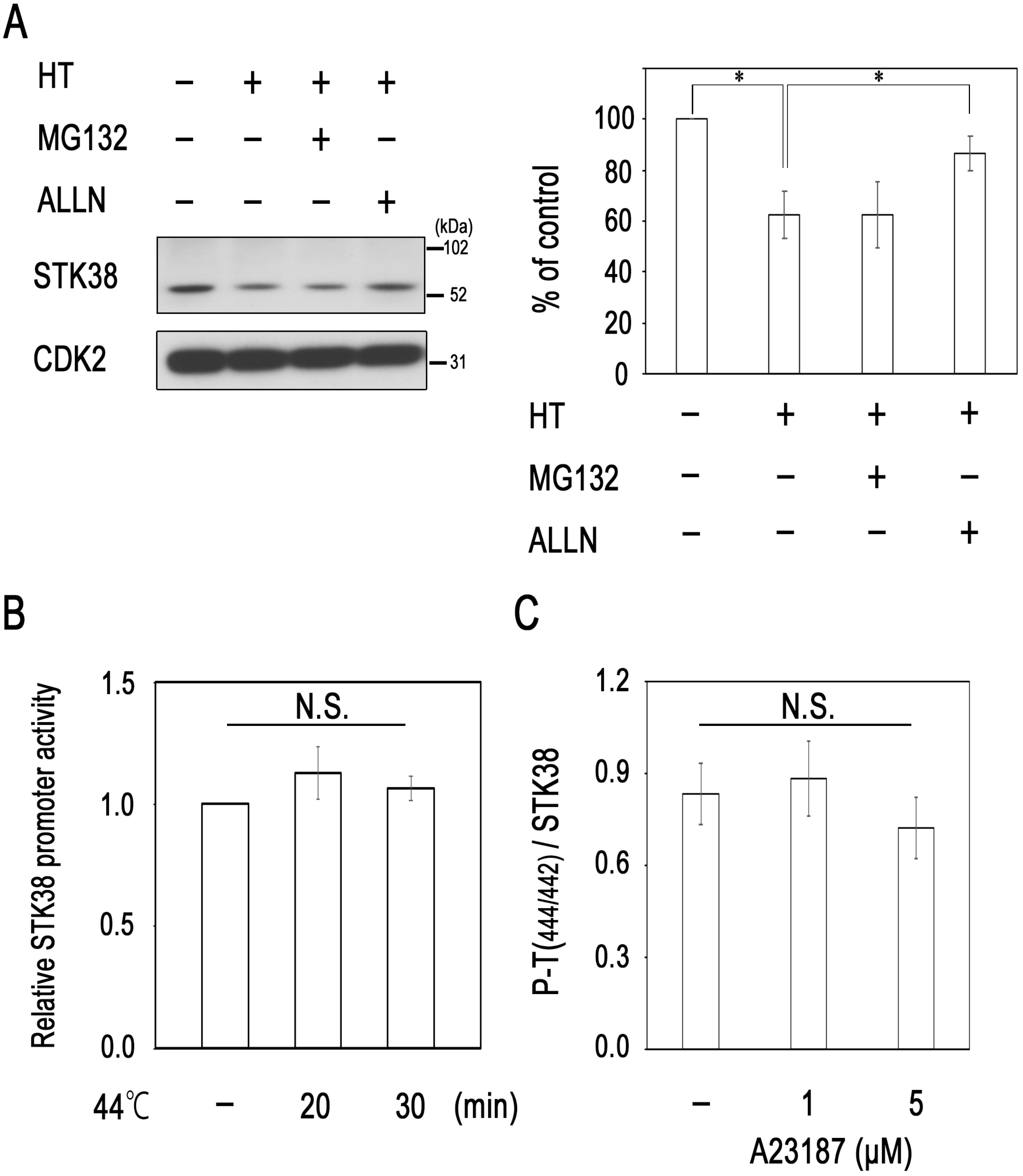
A
HT
MG132
ALLN
(kDa)
102
52
31
STK38
CDK2
% of control
100
80
60
40
20
0
*
B
Relative STK38 promoter activity
1.5
1.0
0.5
0.0
N.S.
44℃
20
30
(min)
C
P-T(444/442) / STK38
1.2
0.9
0.6
0.3
0.0
N.S.
1
5
A23187 (μM)

Supplementary Figure S1. Heat- or A23187-induced degradation of STK38 protein.

(A) LU99 cells were pretreated with DMSO, 10 μM MG132, or 10 μM ALLN for 1 h and then heated to 44 °C for 20 min. Cell lysates were prepared and analysed by western blot with the antibodies against the indicated proteins. CDK2 was used as loading control. Relative levels of STK38 were determined from the western blot by using Image J software. Data are presented as the mean ± standard deviation of three independent experiments. Statistical significance was determined by the Student's *t*-test (*$P < 0.05$). HT, Heat treatment. (B) Luciferase reporter plasmids containing human *STK38* promoter were transfected into COS-7 cells. Twenty-four hours after transfection, the cells were heated to 44 °C for 20, 30 min or left untreated. Then, luciferase assays were performed on cell lysates. Data obtained from three independent experiments are expressed as the mean ± SD of *Firefly* luciferase luminescence values normalised to those of *Renilla.* (C) Ratios of phosphorylated Thr444/Thr442 to total STK38 protein levels were determined from the western blot (Fig. 2B) by using Image J software. Data are presented as the mean ± standard deviation of three independent experiments. Statistical significance was determined by the Student's *t*-test. N. S., not significant.

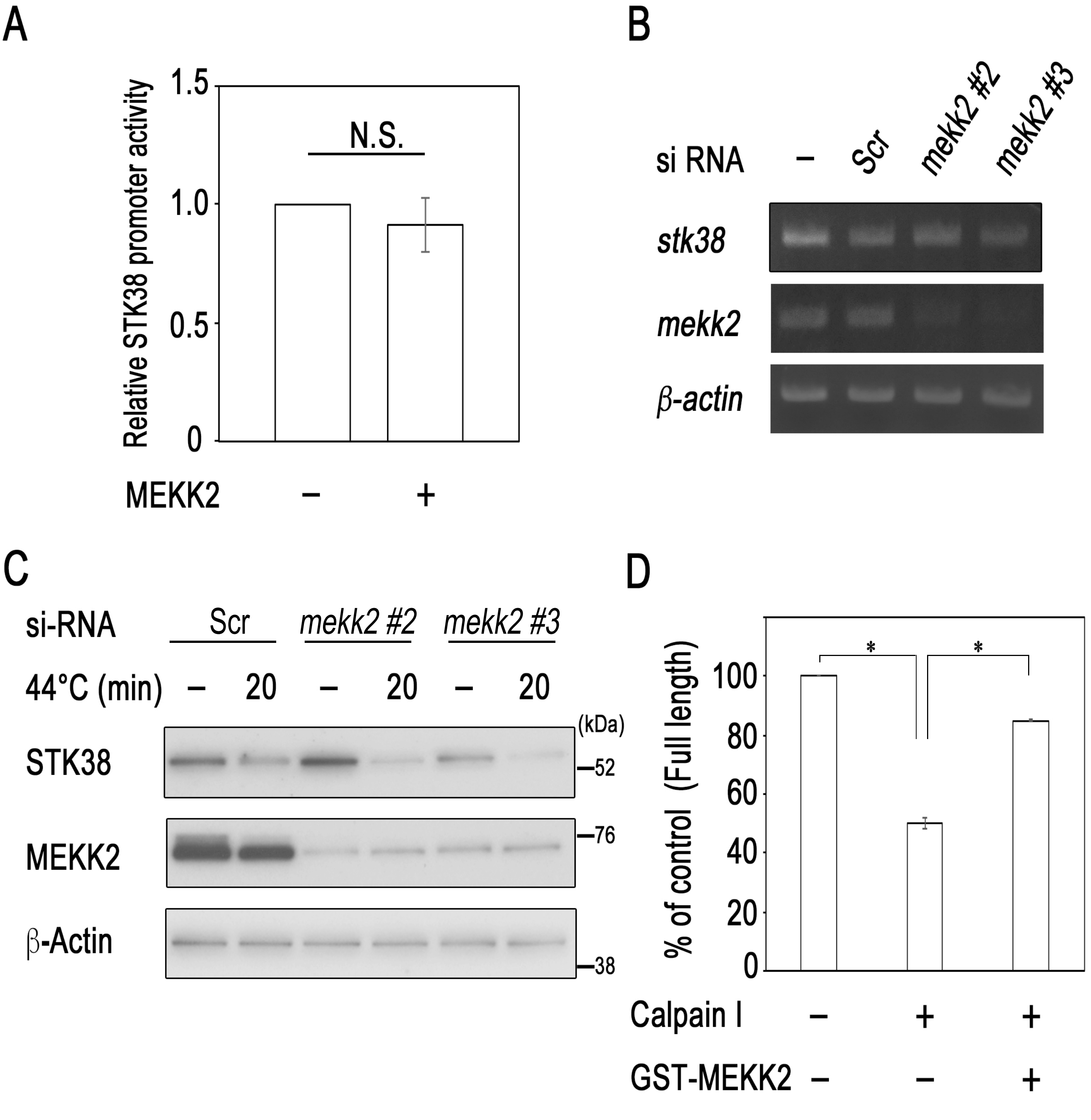
A
Relative STK38 promoter activity
1.5
1.0
0.5
0
N.S.
MEKK2
–
+
B
si RNA
–
Scr
mekk2 #2
mekk2 #3
stk38
mekk2
β-actin
C
si-RNA
Scr
mekk2 #2
mekk2 #3
44°C (min)
–
20
–
20
–
20
(kDa)
STK38
52
MEKK2
76
β-Actin
38
D
% of control (Full length)
100
80
60
40
20
0
*
*
Calpain I
–
+
+
GST-MEKK2
–
–
+

Supplementary Figure S2. MEKK2 regulates STK38 protein expression levels.

(A) Luciferase reporter plasmids containing human *STK38* promoter were transfected with or without FLAG-MEKK2 into COS-7 cells. Twenty-four hours after transfection, luciferase assays were performed on cell lysates. Data obtained from three independent experiments are expressed as the mean ± SD of *Firefly* luciferase luminescence values normalised to those of *Renilla.* Statistical significance was determined by the Student's *t*-test. N. S., not significant.

(B, C) HeLa cells were transfected with the scramble oligonucleotides control (scr) or *MEKK2*-specific siRNA. Forty-eight hours after transfection, the cells were heating at 44 °C for 20 min or left untreated as controls and harvested. Total RNA was prepared from untreated cells, and expression levels of *STK38, MEKK2,* or *ACTB* (β-actin) were estimated by semi-quantitative RT-PCR. Cell lysates were analysed by western blotting with the indicated antibodies. (D) GST-STK38 was incubated with calpain I (0.07 units of calpain I in each lane) in the absence or presence of GST-active MEKK2 for 15 min at 30 °C. *In vitro* reaction products were analysed by western blotting with an anti-STK38 antibody against the indicated proteins. Relative levels of full-length GST-STK38 were determined from the western blot (Figure 3E) using Image J software. Data are presented as the mean ± standard deviation of three independent experiments. Statistical significance was determined by the Student's *t*-test (*$P < 0.05$).

(A)

Start - End	Peptide	Mass	*m/z*	Charge (*z*)	ppm	RT (min)	Phosphorylation Site	Scan #
72 - 78	KET(+79.97)EFLR	1001.4583	501.7367	+2	0.5	23.9	T74	7370
84 - 93	LGLEDFES(+79.97)LK	1229.5580	615.7855	+2	-1.2	43.4	S91	13284
240 - 247	AHRT(+79.97)EFYR	1158.4971	387.1730	+3	0.1	18.0	T243	5040
267 - 273	KAET(+79.97)WKR	997.4746	499.7448	+2	0.5	13.7	T270	3243

(B)

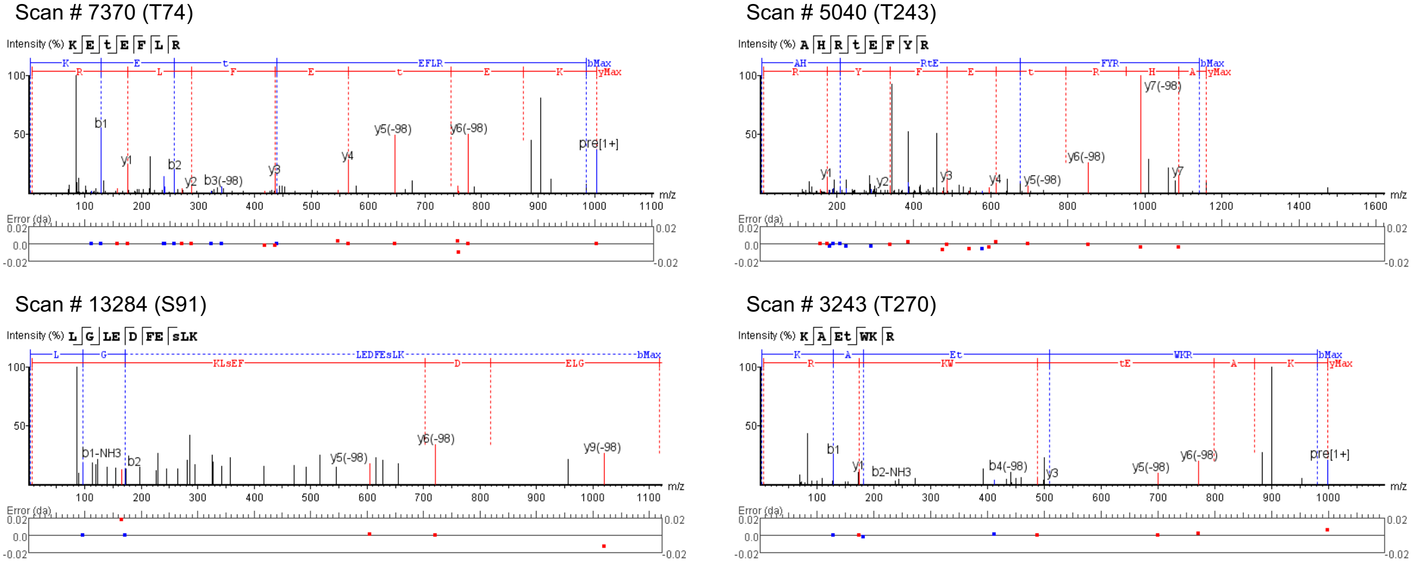

(C)

T74
m/z 501.7366 (*z*=+2)
KET(+79.97)EFLR [72-78]

S91
m/z 615.7858 (*z*=+2)
LGLEDFES(+79.97)LK [84-93]

T243
m/z 387.1736 (*z*=+3)
AHRT(+79.97)EFYR [240-247]

T270
m/z 499.7448 (*z*=+2)
KAET(+79.97)WKR [267-273]

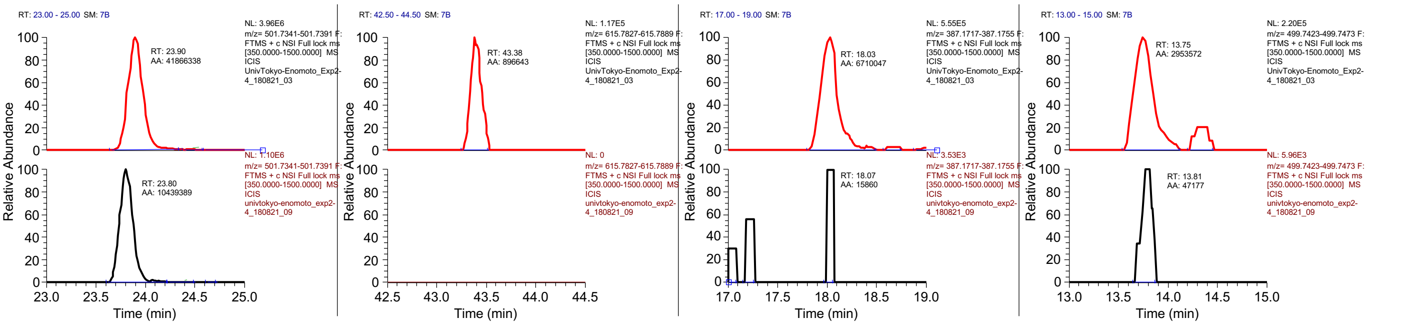

(D)

Phosphorylation Site	*m/z*	Charge (*z*)	Peak area (Ave ± SD, n=4) Kinase (+)	Peak area (Ave ± SD, n=4) Kinase (−)	Area ratio Kinase (+)/(-)
T74	501.7366	+2	44106631 ± 14781408	15323441 ± 5818562	2.9
S91	615.7858	+2	349235 ± 389477	0 ± 0	-
T243	387.1736	+3	8293246 ± 3827981	73883 ± 66311	112.2
T270	499.7448	+2	2188298 ± 600253	37105 ± 12795	59.0

Supplementary Figure S3. Identification of phosphorylated peptides derived from GST-STK38 by nanoLC-MS/MS analysis Identification of phosphorylated peptides derived from GST-STK38 by nanoLC-MS/MS analysis. List (A), MS/MS spectra (B), extracted ion chromatogram (XIC) (C), and XIC peak area (D) of phosphorylated peptides.

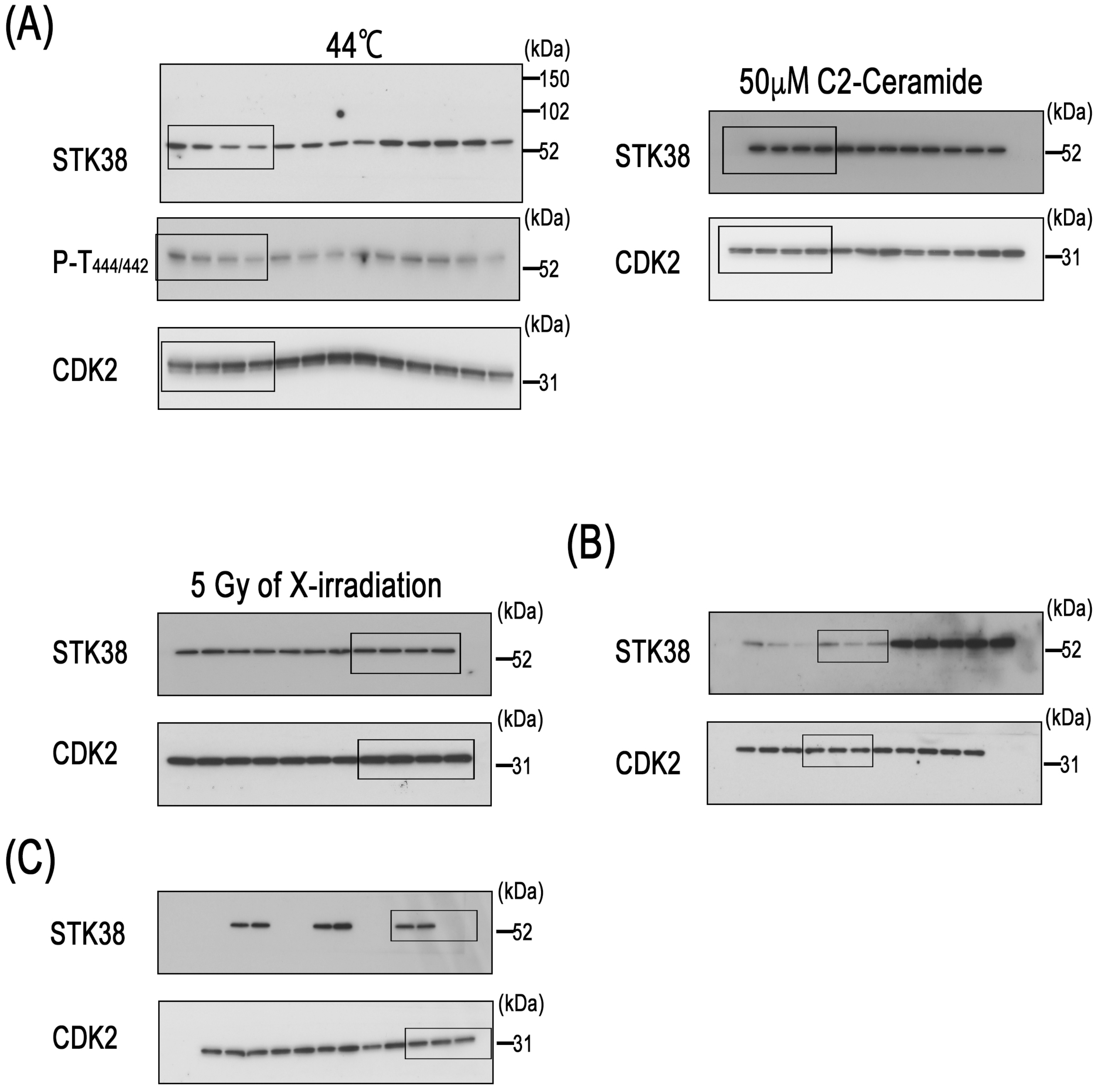

Supplementary Figure S4. Full-length blots corresponding to Figure 1. Different areas of the membrane are separated by white space.

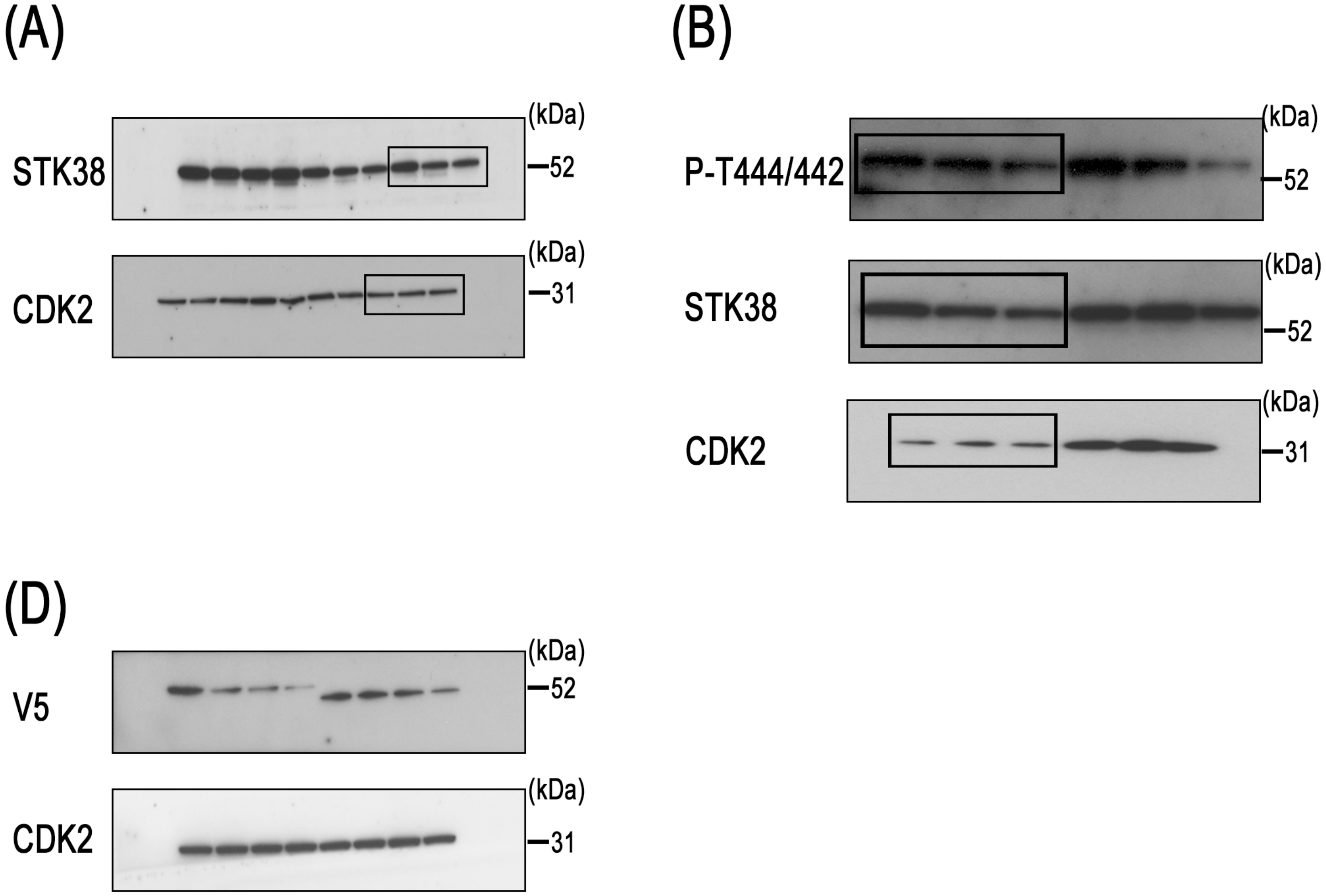

Supplementary Figure S5. Full-length blots corresponding to Figure 2. Different areas of the membrane are separated by white space.

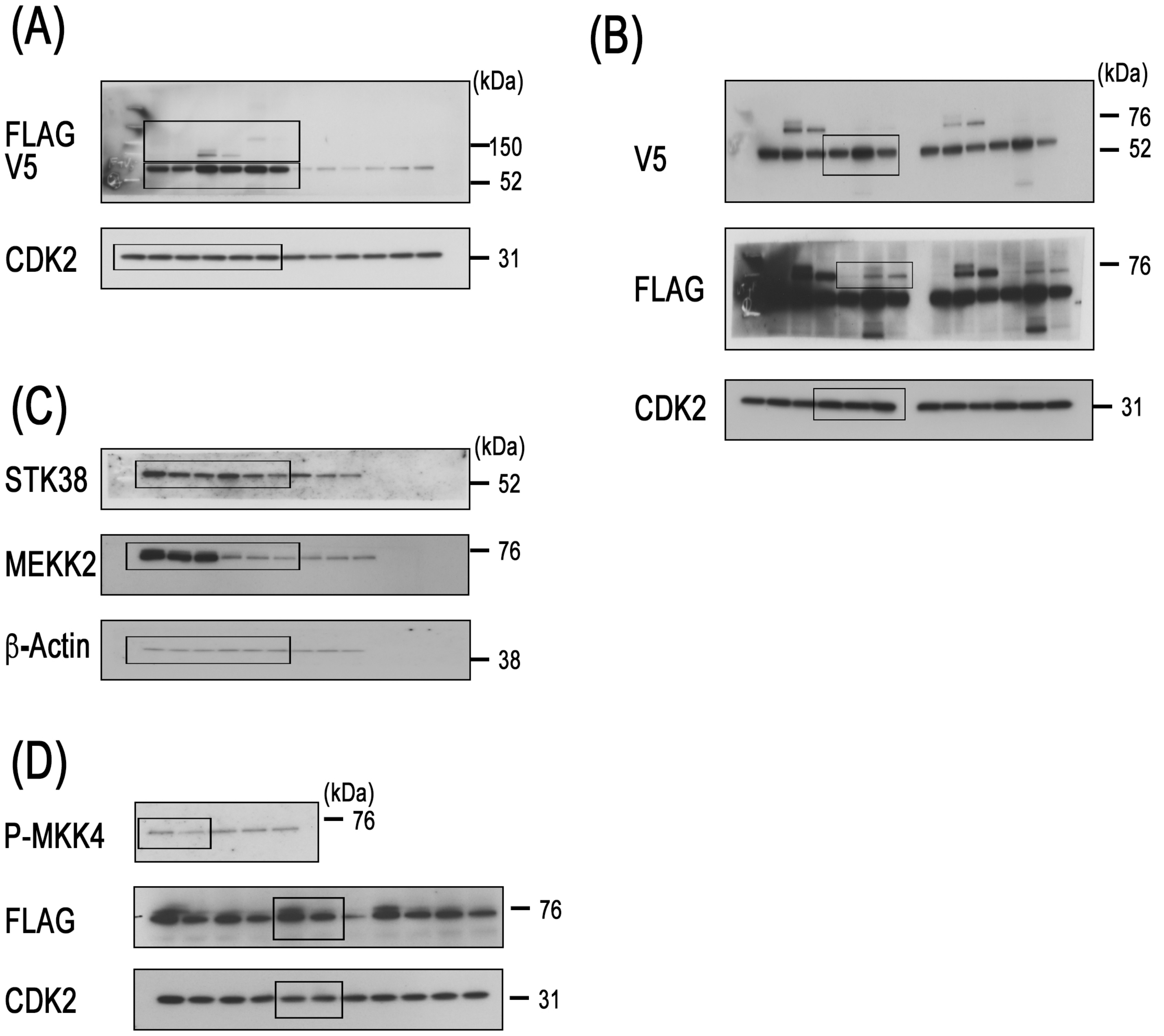

Supplementary Figure S6. Full-length blots corresponding to Figure 3. Different areas of the membrane are separated by white space.

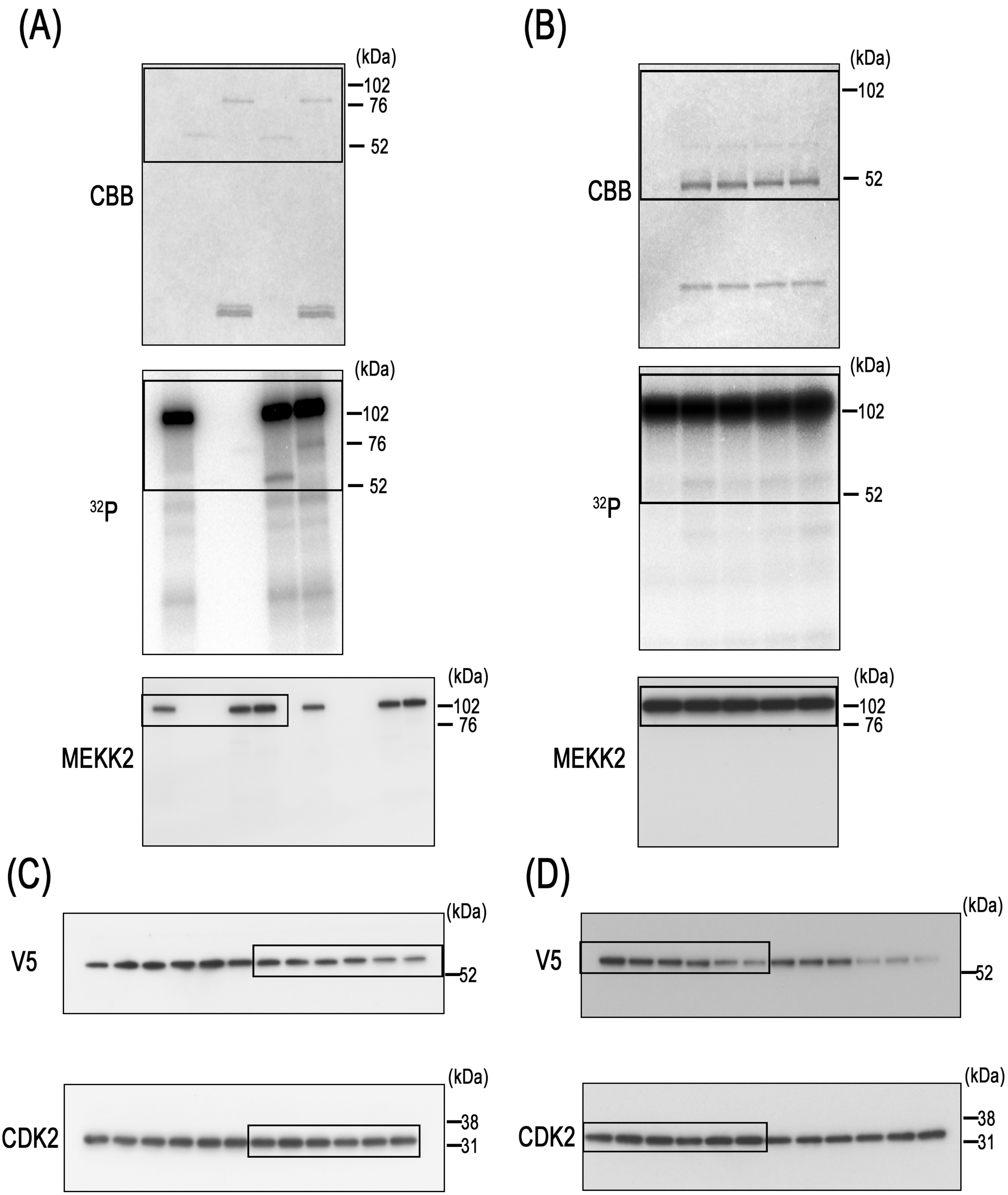

Supplementary Figure S7. Full-length blots corresponding to Figure 4. Different areas of the membrane are separated by white space.
